# Selection and characterization of specific nanobody against bovine virus diarrhea virus (BVDV) E2 protein

**DOI:** 10.1371/journal.pone.0178469

**Published:** 2017-06-05

**Authors:** Tiansen Li, Meiling Huang, Hongran Xiao, Guoqi Zhang, Jinhua Ding, Peng Wu, Hui Zhang, Jinliang Sheng, Chuangfu Chen

**Affiliations:** 1 College of Animal Science and Technology, Shihezi University, Shihezi, Xinjiang, China; 2 Shihezi University Library, Shihezi University, Shihezi, Xinjiang, China; 3 College of Life Science, Shihezi University, Shihezi, Xinjiang, China; Consiglio Nazionale delle Ricerche, ITALY

## Abstract

Bovine viral diarrhea-mucosal disease (BVD-MD) is caused by bovine viral diarrhea virus (BVDV), and results in abortion, stillbirth, and fetal malformation in cows. Here, we constructed the phage display vector pCANTAB 5E-VHH and then transformed it into *Escherichia coli* TG1-competent cells, to construct an initial anti-BVDV nanobody gene library. We obtained a BVDV-E2 antigen epitope bait protein by prokaryotic expression using the nucleotide sequence of the E2 gene of the BVDV-NADL strain published in GenBank. Phage display was used to screen the anti-BVDV nanobody gene library. We successfully constructed a high quality phage display nanobody library, with an initial library capacity of 4.32×10^5^. After the rescue of helper phage, the titer of the phage display nanobody library was 1.3×10^11^. The BVDV-E2 protein was then expressed in *Escherichia coli* (DE3), and a 49.5 kDa band was observed with SDS-PAGE analysis that was consistent with the expected nanobody size. Thus, we were able to isolate one nanobody that exhibits high affinity and specificity against BVDV using phage display techniques. This isolated nanobody was then used in Enzyme Linked Immunosorbent Assay and qRT-PCR, and ELISA analyses of BVDV infection of MDBK cells indicated that the nanobodies exhibited good antiviral effect.

## Introduction

Bovine viral diarrhea-mucosal disease (BVD-MD), also known as bovine viral diarrhea-mucosal disease virus or mucosal virus, is a contagious disease that often occurs in domestic animals[1, 2]. BVD-MD is caused by BVDV viral infection from viruses in the Pestivirus genus of the Flaviviridae family. Infection presents many symptoms for livestock, including diarrhea, persistent infection, miscarriage, and fetal malformation[3]. However, the main pathological features are gastrointestinal mucosal inflammation, intestinal wall erosion and lymphoid tissue necrosis, weight loss and leukopenia. Diseased livestock are the main infection reservoir and transmission to healthy livestock can occur by direct or indirect contact[4]. After invasion of the alimentary and respiratory tracts of susceptible cattle, the virus replicates in the mucosal epithelium and is then transmitted into blood, resulting in viremia[5]. Virus can then be transmitted through the blood and lymph into the lymphoid tissue, leading to lymph node necrosis. Further, the bone marrow granulocyte system is inhibited, which causes leukopenia that stimulates the mononuclear-macrophage system, and mononuclear cell proliferation[6]. Lastly, viral replication during proliferation in the digestive tract mucosal epithelia leads to tissue proliferation, necrosis, and mucosal erosion. Recently, BVDV infections have exploded in number and have reached epidemic levels without effective means of treatment or prevention due to the complexity of the disease pathogenicity[7, 8].

The genome of BVDV is about 12.2–12.5 kbp in length including the 5’ and 3’ UTR and a large open reading frame (ORF) that is present in the genome and comprises four structural proteins (C / P14, E0 / gp48, E1 / gp25, E2 / gp53) and eight kinds of non-structural proteins (P20 / Npro, P7, P125 / (NS2-3 / NS3), P10 / NS4A, P30 / NS4B, P58 / NS5A, P75 / NS5B)[9, 10]. The BVDV-E2 protein is an envelope glycoprotein that is the main site of virus and host cell recognition and adsorption, contains the major antigenic determinants and includes more diversity than any other viral protein[11, 12]. The BVDV-E2 protein induces antibodies that can neutralize the virus with specific targeting[13] and can mediate immune neutralization reaction. E2 proteins are integral in the assembly of viral RNA and particles and in the interaction between viruses and host cells. Due to its plasticity, BVDV can readily adapt to its environment in order to achieve survival in cells. This capacity to adapt is a major cause of loss of efficacy in some vaccines and drugs[14, 15]. The virus has a strong ability to penetrate the tissues and has high binding affinities for target organs and traditional antibodies or drugs have limited ability to control BVDV. Moreover, the virus can reach sites not accessible to conventional antibodies[16].

Nanobodies (VHH) are antibodies that are only found in camelids and some cartilaginous fish[17]. Compared to conventional antibodies, nanobodies are smaller and are equivalent to the heavy chain variable region of typical antibodies[18]. The diameter and length of VHH crystals are only about 2 and 4.5 nm, respectively, making them the smallest functional antibody fragments known[19]. Nanobodies consist of three regions that determine complementarity; the longest being CDR3[20]. The CDR3 region may forms a protruding ring structure that is critical for its binding complementary[21]. and is significantly longer than typical variable regions indicating a superior binding ability compared to other antibodies[22]. Nanobodies are also easy to humanize, and a number of successes have been reported to this effect [23]. Importantly, E2 can cause the production of neutralizing antibodies, which is of relevance in the development of new vaccines, and particularly against E2 proteins[24]. In addition, BVDV-E2 protein can participate in the immune response, and it is also the main cell component used for identification and adsorption between viruses and host cells. Here, we constructed a BVDV nanobody library and screened the nanobodies with BVDV-E2 protein that could provide critical research material for the development of BVDV treatments.

## Materials and methods

### Ethics statement

A one-year-old male camel was obtained from the Manasi Yuanyichang Farm (Manasi state, Changji, Xinjiang, China). During experimentation, the animal had free access to clean water and food along with sufficient space to move. Good living conditions were maintained to ensure a high level of comfort. The animal exhibited healthy and appropriate behavior, comfort and did not display any signs of disease. After experimentation, the animal was intravenously injected with sodium pentobarbital at three times the anesthetic dose in order to induce euthanasia. After death, the animal was then transported to an incinerator for cremation. All efforts were made to minimize animal suffering and the study was approved by the Institutional Committee of Post Graduate Studies and Research at Shihezi University, China.

### Virus, strains, and cells

BVDV was obtained from the China Institute of Veterinary Drug Control (Beijing, China). *Escherichia coli* strain DH5α was grown on Luria-Bertani (Difco, Becton Dickinson) plates or in broth overnight at 37°C with or without ampicillin (50 mg/liter). *E*. *coli* strain TG1 and *E*. *coli* strain 2667 were grown on SOC plates and in broth with and without ampicillin (50 mg/liter) or on 2×YT plates/broth with and without glucose and ampicillin (50 mg/liter) overnight at 37°C. Madin Darby Bovine Kidney (MDBK) cells were purchased from Cell Resource Center, IBMS, CAMS/PUMC (Beijing, China) and was maintained at 37°C in a 5% CO_2_ atmosphere in Dulbecco modified Eagle medium (DMEM) (Gibco, U.S.) containing 10% fetal bovine serum (FBS) (Gibco, U.S.). Cells were plated in 6-well tissue culture plates (Nalge Nunc International, Naperville, and III).

### Camel immunization

A healthy young male camel (*Camelus bactrianus*) was immunized six times a week with 125 g of BVDV virus that was resuspended in PBS in the presence of an equal volume of complete Freund’s adjuvant (Sigma-Aldrich, U.S.). Serum antibody titers were determined using the agar diffusion method. The camel was then immunized once a week with antigen in the presence of incomplete Freund’s adjuvant (Sigma-Aldrich, U.S.). Five days after the last injection, peripheral blood mononuclear lymphocytes (PBMLs) were extracted from 100 ml of blood sample.

### Phage library construction

For phage library construction, total mRNA was extracted from the PBMLs, and then the VHH genes were amplified using nested PCR. The PCR protocol consisted of an initial denaturation step at 98°C for 10 s, followed 20 cycles of 50°C for 20 s, 72°C for 1 min and then 40 additional cycles of 98°C for 30 s, 68°C for 1 min, and 72°C for 10 min followed by a final 16°C hold. The PCR products (~700bp) were purified using agarose gel electrophoresis and used as templates for a second PCR that consisted of 98°C for 10 s, followed by five cycles of 50°C for 20 s and 72°C for 40 s and an additional 35 cycles of 98°C for 310 s, 72°C for 40 s, and 72°C for 10 min followed by a final hold at 16°C. The final purified PCR products (~450 bp) and the pCANTAB 5E vector (Xinjiang University, China) were digested with *Sfi* I, then ligated by with T4 DNA ligase, and electro-transformed into competent *E*. *coli* TG1 cells. Transformants were grown in 2*TY medium containing 2% glucose and 100 μg/ml ampicillin at 37°C overnight.

### Cloning, expression, and purification of BVDV-E2 proteins

The E2 ORF was amplified by PCR from the cDNA of BVDV. The amplified DNA fragment was then cloned into the pGEX-4T-1 vector (Novagen, Madison, WI, U.S.) to generate the recombinant plasmid pGEX-4T-E2, and expressed in *E*. *coli* BL21 (DE3) as an N-terminally GST-tagged fusion protein. The expression of the recombinant protein was analyzed by 12% SDS-PAGE. Recombinant BVDV-E2 protein was purified as described previously[25].

### Identification of recombinant BVDV-E2 through Western blot

Cell lysates containing recombinant E2 protein were analyzed using the Western blot method, as previously described[26]. Briefly, the purified recombinant E2 protein was separated by 15% SDS-PAGE. Proteins were then transferred to nitrocellulose by semi-dry Western blotting for 40 min in transfer buffer. Membranes were incubated in blocking solution (5% nonfat milk in TBST) for 1 h at room temperature. The membrane was then incubated at room temperature for 1 h with BVDV immunized camel serum, diluted to 1:500 in 2.5% milk/TBST. After three washes, the membrane was incubated with peroxides conjugated sheep anti-camel-HRP conjugated antibody for 1 h at room temperature in 5% milk/TBST. After three washes, bound conjugate was visualized with an enhanced HRP-DAB substrate color kit (Tiangen Biotech Beijing, China). Western blotting was performed in triplicate.

### Panning of special VHH against BVDV E2

The VHH phage display library was panned for three consecutive rounds. A 96-well plate was coated with 100 μl toxicity BVDV that was resuspended and diluted in carbonate buffer (pH 9.6) overnight at 4°C. The wells were then washed three times with 300 μl 1% PBST and blocked with 200 μl of 2% skim milk and incubated for 2 h at 37°C. *E*. *coli* TG1 containing the phage library was then added to the wells and incubated with rotation at 150 rpm for 30 min (TG1 was incubated at 37°C for 2 h and mixed it with 300 μl 2% PBSM). Wells were then washed three times with 300 μl 1% PBST and 10 times with 300 μl 1 M PBS (washing times were increased in the second and third rounds to reduce non-specific binding), and Two-hundred μl of *E*. *coli* 2667 (OD = 0.6) was then added to the wells and incubated for 30 min at 37°C followed by addition of 100 μl glycine (pH 2.7) and further incubation at 37°C for 10 min. The mixture was then placed in 1.5 ml Eppendorf tubesand mixed with 20 μl 1 M tris (pH 9.1) and then infected with TG1 (that were pretreated) and incubated at 37°C for 30 min. Panning output was assessed on the following day, and the phage library was collected and amplified with targets selected from the first to the third rounds of panning.

### Preliminary identification the positive recombinant antibodies by phage ELISA

A 96-well plate was coated overnight with 200 μl of inactivated BVDV that was resuspended and diluted in carbonate buffer (pH 9.6) and incubated at 4°C overnight. M13K07 was used as a positive control and PBS as the negative control. All wells were blocked with 200 μl of 2% skim milk, followed by incubation at 37°C for 1 h and three washes with 1 M PBS. One-hundred and sixty μl of monoclonal VHH-phage was mixed with 40 μl 2% PBSM before use. The VHH-phage that had been pretreated at 37°C for 2 h was then added and allowed to bind with anti-M13-HRP (1:5000 dilution with 2% PBS) and washed three times. Then, 100 μl of TMB was added to the wells and incubated at 37°C for 30 min in the dark. Fifty μl of 2 M H_2_SO_4_ was then added to end the reaction. The optical density (OD) was measured using an ELISA micro plate reader at a 492 nm wavelength.

### Detection of specific antibodies for BVDV-E2 with indirect ELISA

Specific phage clones were identified by indirect ELISA using the anti-E-tag-HRP antibody. Nine individual colonies from the third rounds of panning were selected, and nanobodies were expressed in the periplasmic space of log-phase *E*. *coli* TG1 with 1 mM IPTG (isopropyl D-1-thiogalactopyranoside). The fusion nanobody was extracted using osmotic shock and detected by anti-E-tag-HRP antibody (1:5000 dilution). DNA from positive clones was then sequenced to identify unique nanobody genes.

### Cloning, expression, and purification of the nanobody

Nanobody fragments were amplified with the primers VHH-F: 5’- *GAATTC*AGTTGCAGCTCGTGGAGTCTGG-3’ (*EcoR* I) and VHH-R: 5’- *AAGCTT*TGCGGCACGCGGTTCCA-3’ (*Hind* III) and then cloned into pGEX-4T-1 vectors after double digestion with restriction enzymes *EcoRI* and *Hind III*. The recombinant plasmids were transformed into *E*. *coli* BL21 (DE3) and incubated at 37°C. The cultures were induced using isopropyl-b-thiogalactopyranoside (IPTG) when the OD600 ranged from 0.6 to 0.7. The cells were collected through centrifugation and suspended in lysis buffer (pH 8.0) and incubated at 4°C, overnight. The recombinant bacteria were ultrasonically treated (three cycles, 20 min/cycle, working for 5 s, and resting for 5 s) to obtain cell lysates. The proteins of interest were purified using GSTrap^TM^ FF affinity resins (GE, U.S.) and then identified by SDS–PAGE.

### Western blotting

Purified nanobody was separated by 15% SDS-PAGE. For Western blotting, the protein bands were transferred to nitrocellulose membranes and blocked with 4% skim milk for 1 h at 37°C. The membrane was washed three times with PBST and nanobodies were detected using HRP-conjugated anti-GST monoclonal antibody (1:5000 dilution, Cwbiotech, China) and the protein bands were detected with an enhanced HRP-DAB substrate color kit (Tiangen Biotech, Beijing,Co. Ltd., China). Western blotting was performed in triplicate.

### Double nanobodies sandwich ELISA

Sandwich ELISA was used to assess BVDV-E2 specificity of the nanobody. Nanobody was coated in the wells of 96-well microtiter plates, and the plate was incubated overnight at 4°C. BVDV-E2 was added into the corresponding wells. After 2 h of incubation at 37°C, the wells were washed three times with PBST. HRP-conjugated anti-E-tag antibody (1:5000) was used as the secondary antibody. TMB substrate was added and allowed to react for 15 min with incubation at 37°C. The reaction was ended with H_2_SO_4_ addition (50 μl, 2 M). Optical density (OD) was then measured by ELISA using a micro plate reader at a 492 nm wavelength.

### Determination of virus neutralization by the nanobody

Nanobody was mixed with BVDV concentrate at a 2:1 ratio while BVDV immunized camel serum was mixed with BVDV concentrate at a 2:1 ratio (positive control), and PBS was mixed with BVDV concentrate at a 2:1 ratio (negative control). After 2 h of incubation at 37°C, the mixed liquor was found to contain infected MDBK cells. The next day, pathological changes of MDBK cells were observed by microscope.

### BVDV virus copy number assessed with qRT-PCR

The nanobodies were incubated with BVDV for about 2 h, followed by addition of infected MDBK cells, Cells were collected at 48 h and 72 h, and total viral RNA was extracted followed by reverse transcription of cDNA. The absolute quantification method was used to determine the number of BVDV NADL viral copy numbers. Different doses of the VHH nanobody blocking of BVDV replication were then determined SPSS software was used for data processing and analysis.

## Results

### Construction of a nanobody library for BVDV

**Fig 1:**
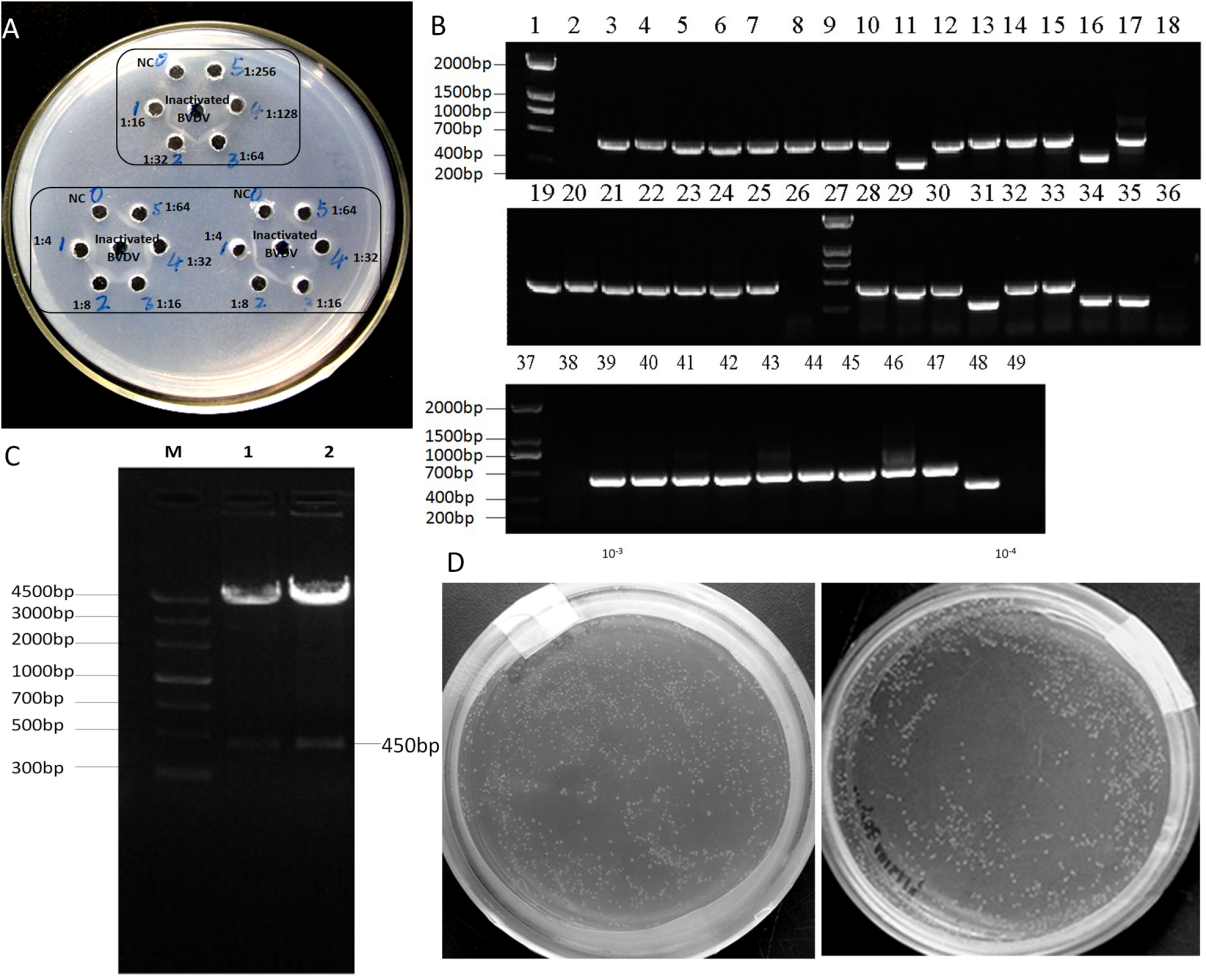
Construction and identification of a BVDV nanobody library. (A) Antibody titer was detected by agar diffusion test. Center bore: inactivated BVDV solution; NC: negative control. Order of antibody dilution set: 1:4, 1:8, 1:16, 1:32, 1:64, 1:128, 1:256. (B) Nested PCR amplification results of VHH and the 450 bp sequences that were obtained. (C) Results of enzyme digestion and identification of pCANTAB5E-VHH M: Marker; 1–2: pCANTAB5E-VHH digested with *Sfi*I. (D) The size of the library (1.3×10^11^ CFU/ml) was determined by counting the number of clones after gradient dilution.

The VHH library was constructed after immunization of a healthy camel with the BVDV virus for 21 weeks. After separation of serum, the results using the 1:64 serum dilutions showed that a good antibody titer had been raised, as indicated by the presence of a clear band (Fig 1A). After nested PCR, a 450-bp gene fragment was obtained (Fig 1B), cloned into the phagemid vector pCANTAB 5E (Fig 1C), and transformed into *E*. *coli* TG1 cells. The initial constructed library contained 4.32×10^5^ colonies. After rescue of the helper phage, the phage titer of nanobody library was 1.3×10^11^ with 96% exhibiting the appropriate size of gene insert (Fig 1D), which suggests that the diversity of the antibody library was good.

### Expression, purification, and Western blot analysis of the BVDV-E2 recombinant protein

**Fig 2:**
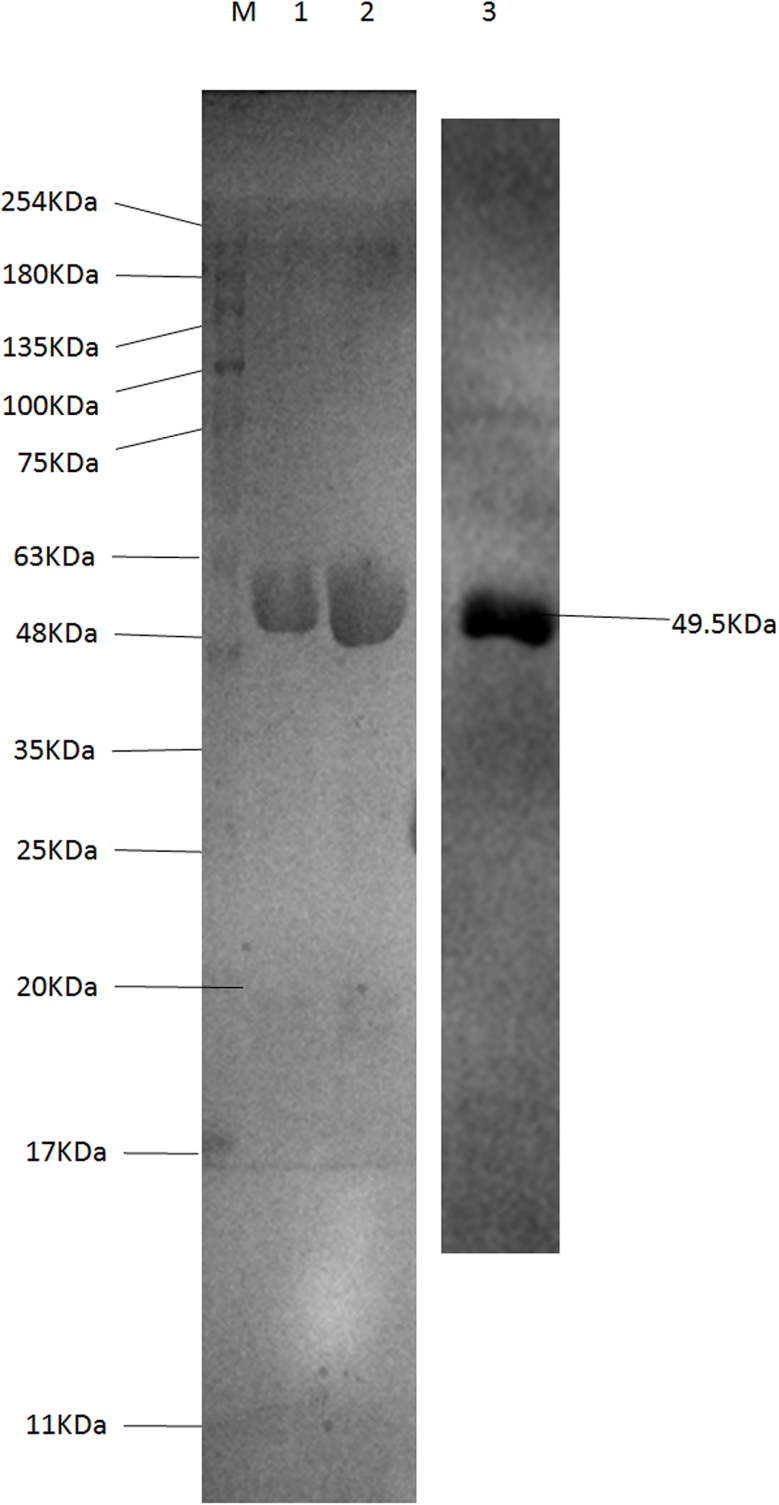
SDS–PAGE analysis of the expression and purification of BVDV-E2 recombinant protein. Expression of BVDV-E2 in *E*. *coli* BL21 (DE3). When DE3 bacteria reached the logarithmic growth phase, IPTG was added to a final concentration of 1 mmol/l, and induced for 16h at 28°C and Western-blot results showed that the strip with the expected protein size of 49.5 kDa was consistent with Lanes 1 and 2 and purification of recombinant BVDV-E2 protein. Lane 3: The Western-blot results of BVDV-E2 protein. Lane M: Molecular weight markers, size indicated in kDa.

The BVDV-E2 recombinant protein was induced and purified by GSTrap™ HP columns. Sodium dodecyl sulfate polyacrylamide gel electrophoresis (SDS-PAGE) analysis then demonstrated the high quality of the BVDV-E2 recombinant protein that was obtained and which exhibited a purity level of over 90%. The BVDV-E2 recombinant protein had the expected molecular weight of 49.5 kDa. The purified E2 recombinant protein was analyzed with 12% SDS–PAGE and confirmed with Western blot, analysis which resulted in the observation of target protein bands (49.5 kDa) on the NC membrane (Fig 2).

### Panning of a BVDV-E2 specific nanobody by phage-ELISA

Recovery rate and enrichment were calculated after three rounds of “absorption–elution–amplification” of specific affinity screening experiments using BVDV-E2 protein as the bait protein. Calculations were performed by recording the input and output of nanobody libraries during each round of panning, Results are provided in Table 1 where Results showed that: Recovery = phage output (CFU)/phage input (CFU), enrichment = next recovery/front recovery and Enriching factors = output/input. pfu: plaque-forming unit (Table 1).

**Table 1:** Selective enrichment of nanobodies from the libraries during panning.

Panning times	Phage input (cfu)	Phage output (cfu)	Recovery rate	Enrichment
1	3.31×10^12^	5.16×10^8^	1.56×10^−4^	—
2	2.11×10^10^	6.49×10^5^	3.08×10^−5^	19.7
3	1.73×10^9^	5.78×10^2^	3.34×10^−7^	108.4

### Preliminary validation results of Phage ELISA

**Fig 3:**
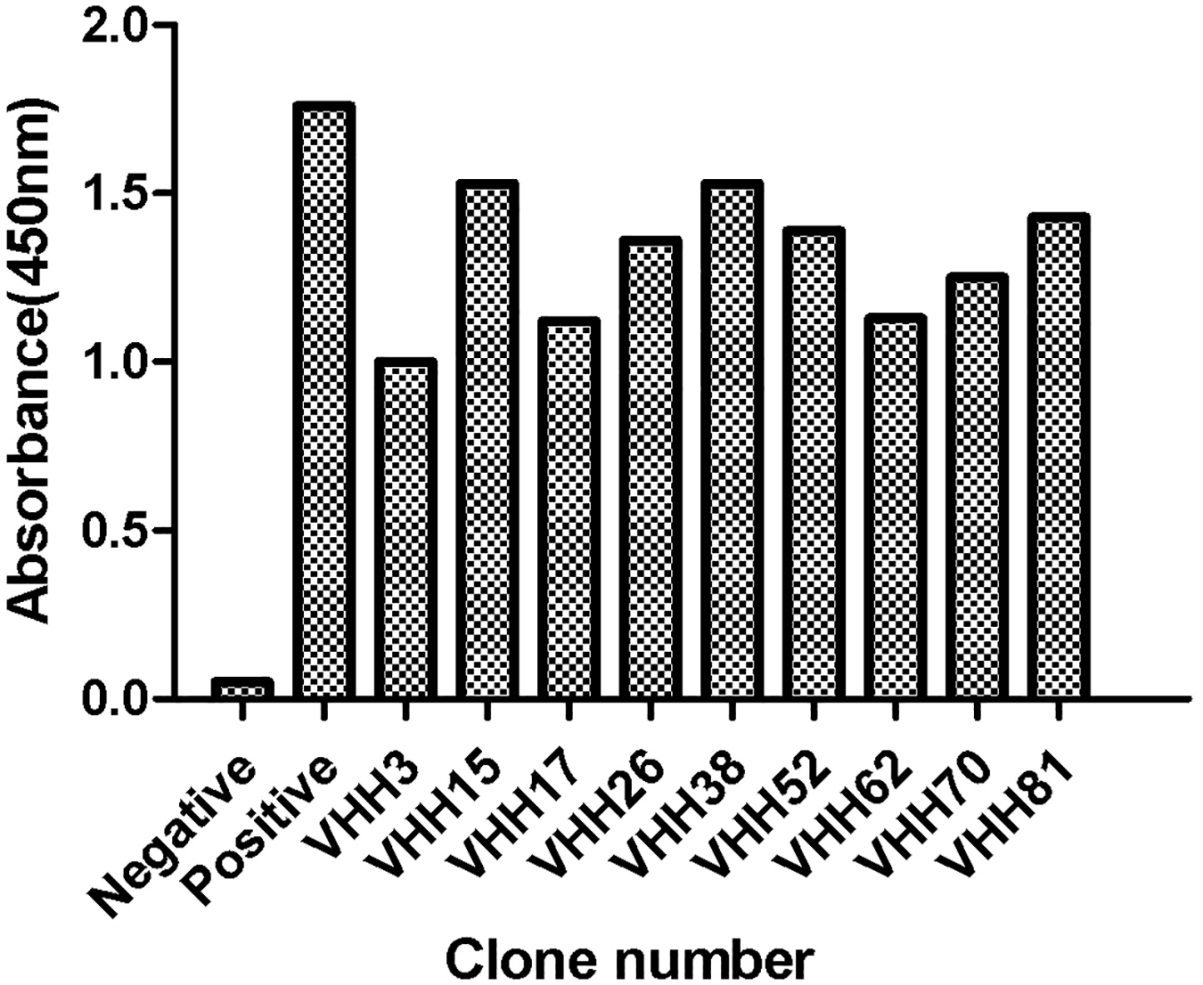
Monoclonal phage ELISA. A total of 96 random clones from the library were analyzed with monoclonal phage ELISA. BVDV-E2 antigens at 10 μg/ml were coated in each well. PBS served as the negative control and M13K07 was used as the positive control. A total of nine clones were selected on the basis of absorbance. The x-axis shows the number of clones, and the y-axis shows the absorbance values at 450 nm.

Three rounds of screening were performed to produce the nanobodies, followed by affinity screening. During screening, 96 single colonies were randomly selected from each round and then screened for BVDV virus recognition by periplasmic extraction followed by ELISA. After the rescue of M13K07 helper phage and preparation of phage nanobody, nine colonies were found to specifically bind to BVDV-E2 (Fig 3).

### Indirect ELISA detection of specific antibody against BVDV-E2

**Fig 4:**
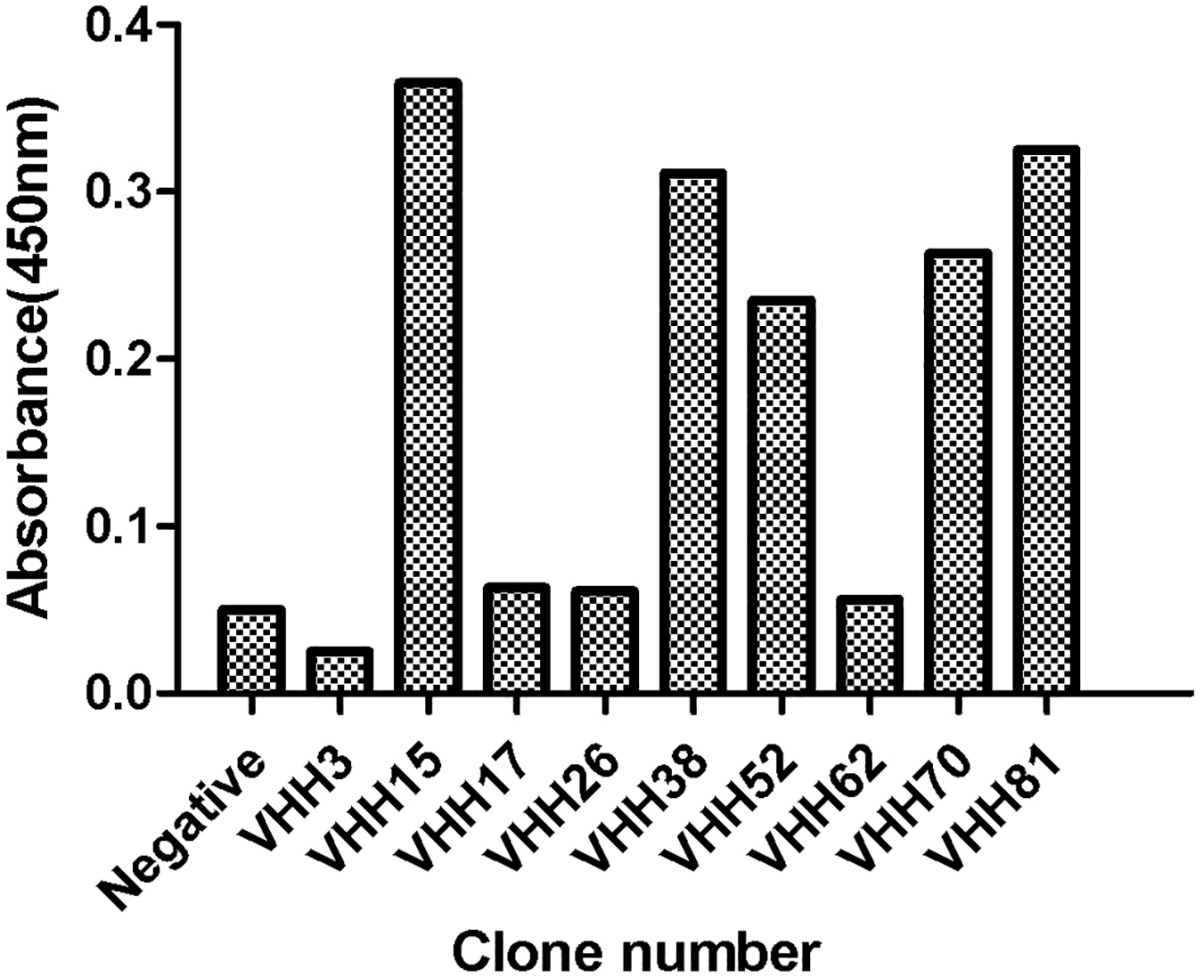
Monoclonal phage ELISA. A total of nine clones (out of 96) were analyzed with monoclonal phage ELISA. BVDV-E2 antigens at 10 μg/ml were coated in each well. PBS served as the negative control. A total of five clones were selected on the basis of absorbance. The x-axis presents the clone number, and the y-axis shows the absorbance values at 450 nm.

**Fig 5:**
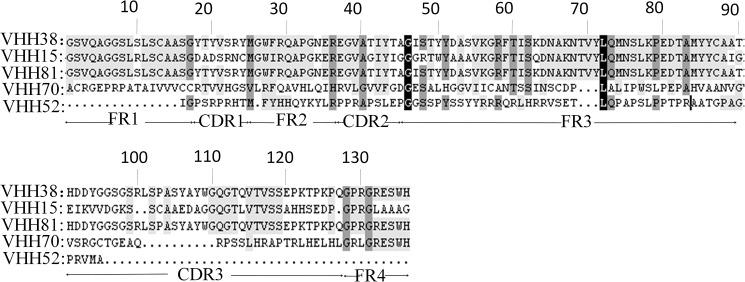
Multiple amino acid sequence alignment of BVDV-E2 nanobody clones. The framework and CDR regions and amino acid numbering were performed as stipulated in Gene.DOC. The CDR regions outlined in lines. The CDR regions are outlined in lines. Sequencing analysis indicated that the nanobody clones were highly homologous to the camel VHH sequence.

Positive clones were induced by IPTG with recombinant soluble VHH nanobodies and again analyzed by indirect ELISA. Results indicated that there were five monoclonal antibodies capable of specifically binding to the BVDV-E2 protein (Fig 4). DNA sequence analysis indicated that the antibodies had close homology with the nanobody sequences belonging to camels (Fig 5). However, their paratope (CDR3 region) amino acid sequences differed somewhat which is shown in Fig 5.

### Expression and purification the nanobody VHH 15

**Fig 6:**
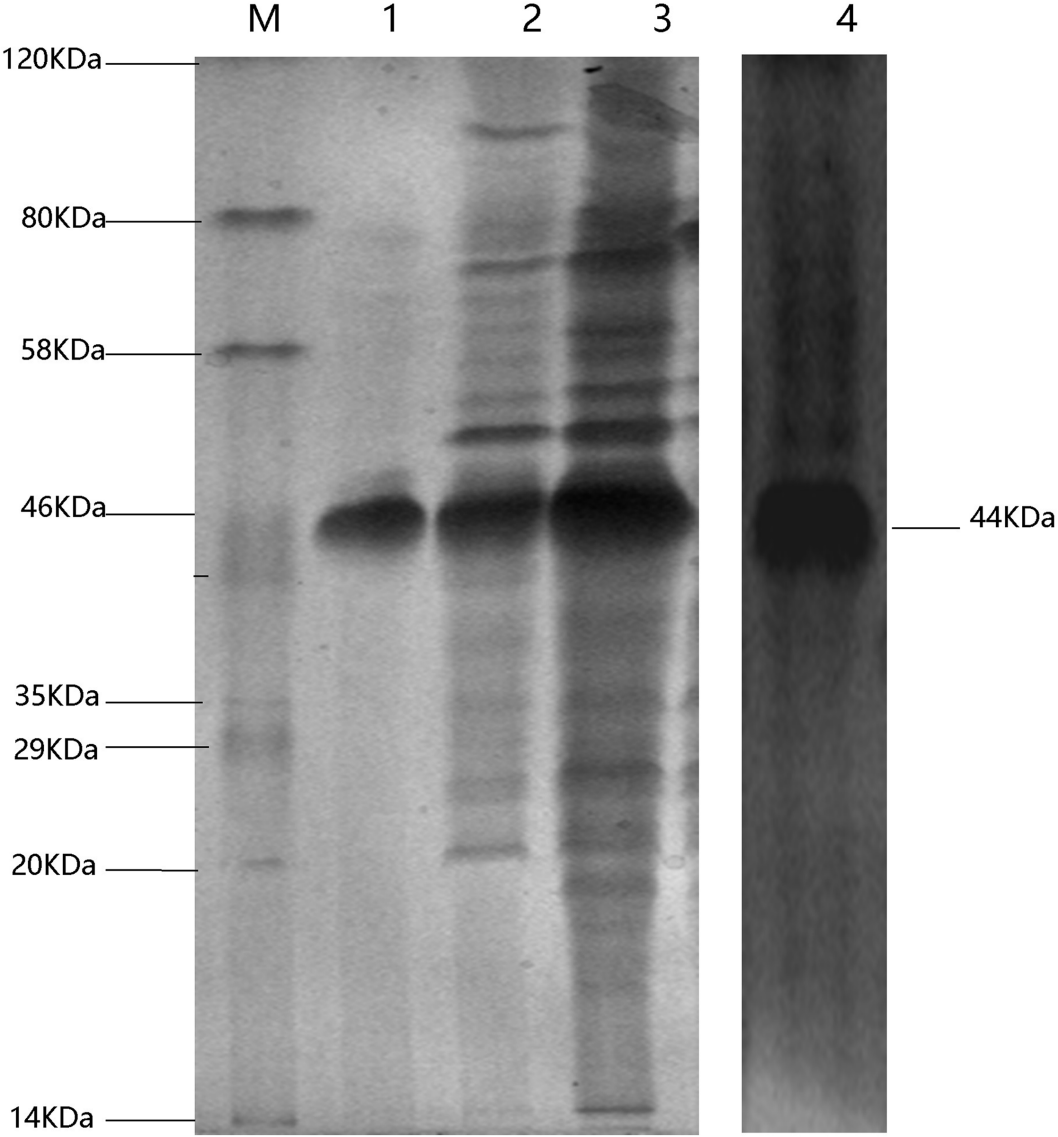
Purification of BVDV-specific nanobody. The nanobody that was encoded by candidate DNA sequence was purified using affinity chromatography. The nanobody was detected using Coomassie brilliant blue staining under SDS-PAGE.

Nanobodies were expressed in *E*. *coli* DE3 cells and then induced and analyzed using SDS-PAGE (12%). The recovered nanobody had the expected molecular weight of 44 kDa (Fig 6). In addition, the purified nanobody recombinant was analyzed using SDS-PAGE (12%), which yielded a single band. Lastly, the nanobody was identified via Western-blot, and a band was observed on the NC membrane (Fig 6).

### Identification of the BVDV-E2 nanobody

**Fig 7:**
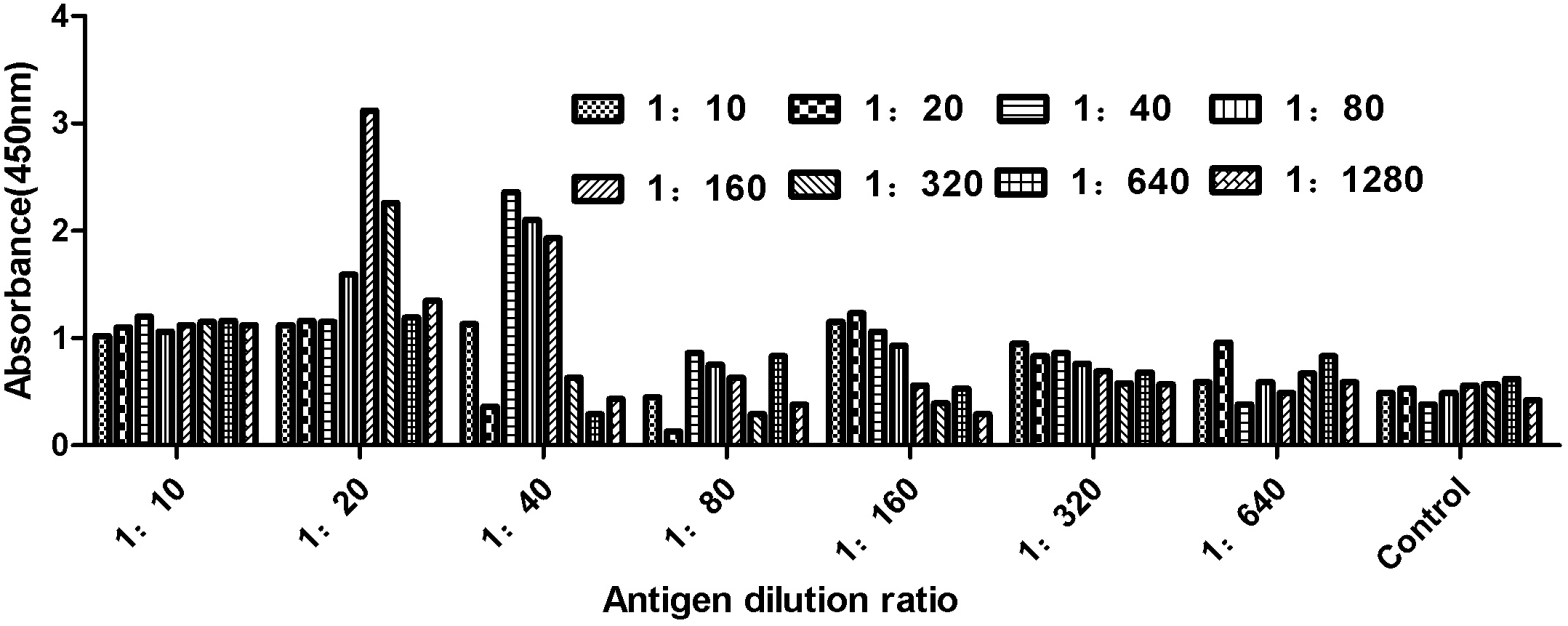
Verification of the binding ability of the nanobody and antigen protein. The optimal nanobody dilution ratio was determined by double antibody sandwich ELISA. When the dilution ratio of antigen was 1:20 and the dilution ratio of nanobody was 1:160 and the binding capacity was the strongest.

The specificity of the nanobody was assessed using ELISA. Results shown in Fig 7 indicated that Nanobody VHH 15 was able to bind specifically to the BVDV virus. Based on these results, VHH 15 was used in a nanobody-pairing assay. A nanobody-pairing assay was performed using this BVDV-E2-specific nanobody. Results indicated that VHH 15 could combine with BVDV virus particles for further diagnostic application based on sandwich ELISA.

### Ability of the nanobody to neutralize the BVDV

**Fig 8:**
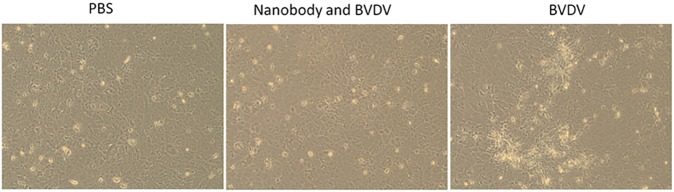
Number of plaques in the field of view. (A) Non-viral plaque of MDBK cells in the uninfected group; (B) BVDV concentrate and nanobody VHH15 were mixed thoroughly in a 15 ml centrifuge tube at a 2:1 ratio and incubated in a 37°C incubator for 60 min. After the nanobody VHH15 was incubated with BVDV, it was added to the MDBK cells, and viral plaques lessened; (C) BVDV concentrate and PBS were mixed thoroughly in a 15 ml centrifuge tube at a 2:1 ratio and incubated in a 37°C incubator for 60 min. BVDV-infected MDBK cells showed significant amounts of viral plaque.

The negative control group exhibited a virus plaque, while no viral plaques were observed in the positive controls and experimental groups. Plaque numbers were calculated using microscopy (Fig 8), and further demonstrated that the recovered nanobody could neutralize BVDV, thus mitigating cellular infection.

**Fig 9:**
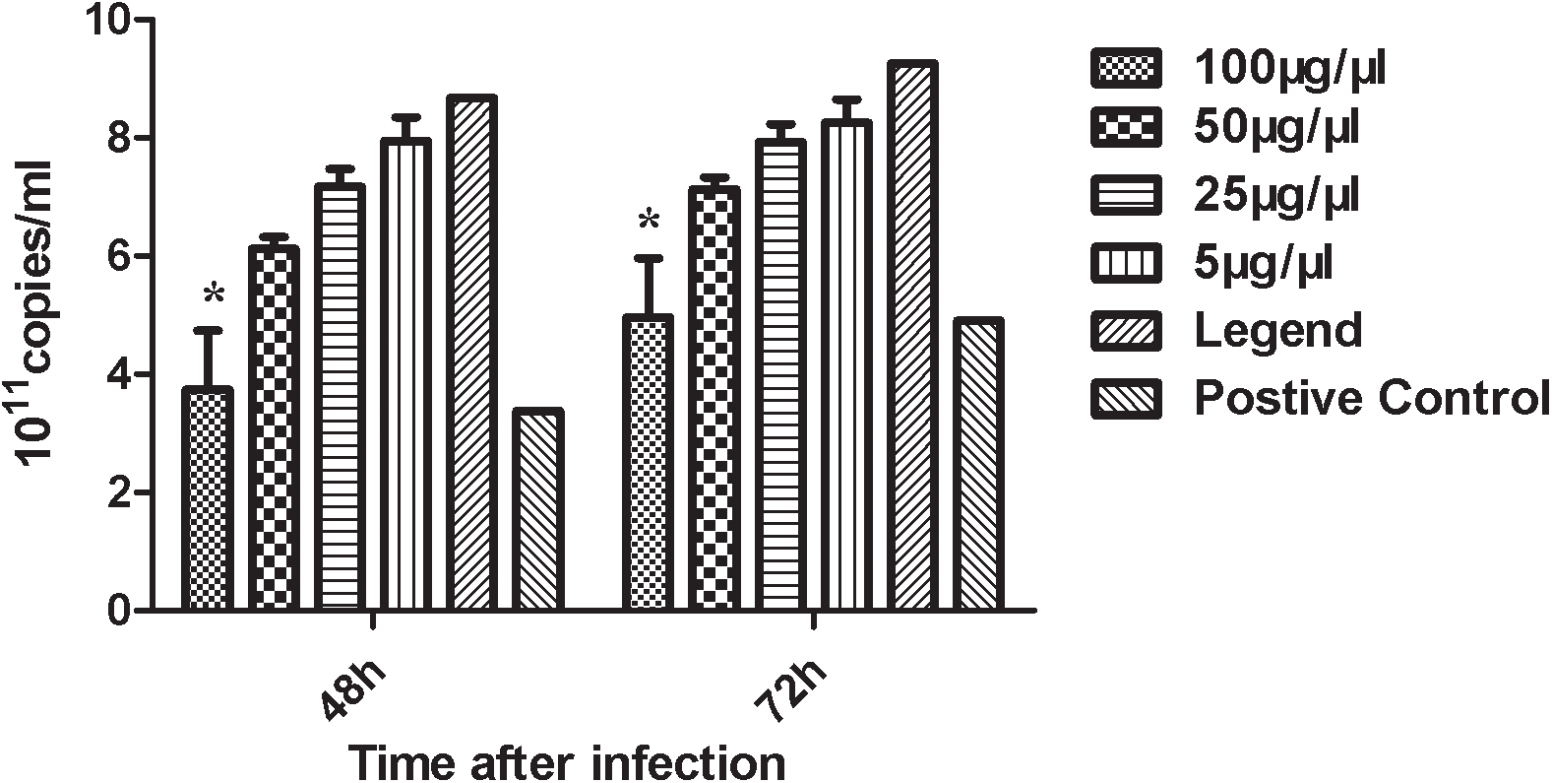
Effect of different doses of VHH nanobodies on BVDV replication copy number. After different doses of nanobody and BVDV were prepared and incubation for 1.5–2 h, the mixture was used to infect MDBK cells. After 48 h and 72 h of incubation, the cells were collected and the total RNA was extracted and reverse-transcribed into cDNA, which was then subjected to qRT-PCR detection.

Viral copy number was calculated for different VHH nanobody groups with different concentrations using the E2 gene standard curve and a copy number formula. qRT-PCR results showed that 100 μg/ml of VHH had a blocking effect on BVDV copy numbers in MDBK cells and thus, demonstrated an ability to neutralize the virus (Fig 9).

## Discussion

In this study, a dromedary immune VHH library was constructed and a panning specific nanobody was generated against BVDV-E2 proteins using a phage display technique. Three biopanning selection procedures were performed on immobilized BVDV-E2. The stringency of the planning procedure was increased to produce an anti-BVDV-E2 nanobody that exhibited high affinity and specificity. In the screening process, a large number of monoclonal phages were enriched. The specific function of the monoclonal nanobody was confirmed using competitive ELISA through interaction of BVDV-E2 protein and phage enrichment products on the surfaces of solid plates. HRP-labeled M13 antibody was used to detect the chromogenic reaction, such that only positive monoclonal antibodies were detected. This process allowed us to greatly reduce the number of the sequences present and increase the success rate of sequencing.

Nanobodies play an important role in the treatment of many diseases[27] and they exhibit efficacy in treatments in the cell nucleus, cytoplasm, and endoplasmic reticulum[28]. In particular, the high specificity and affinity of nanobodies make them valuable tools for neutralizing antigens in cells[29]. Conventional antibodies have been tested as targeting drugs, but development has been limited due to poor stability and the high cost of production[28]. However, nanobodies can overcome those disadvantages and also perform new functionalities[30]. Moreover, nanobodies are the smallest antibody known and can be easily produced in prokaryotic and eukaryotic expression systems[31].

BVDV vaccines are attenuated and inactivated viruses that have shortcomings that consists of safety risks and poor immunogenicity, among others[32]. Although nanobodies exhibit high affinities and specificities, immunogenicity and toxicity are very low and do not adhere as easily as single chain antibodies[33]. However, the treatment and prevention of BVDV still presents a significant challenge because the current mechanism of BVDV infection is not well understood and the mutation rate of BVDV is high[34]. The current vaccine for BVDV is not very effective as a comprehensive control and suffers from security problems, as is typical of other vaccines[35]. Thus, BVDV vaccine research and development should consider the use of new, innovative technologies, methods and ideas in order to solve existing issues with BVDV vaccines. Here, we have demonstrated the utility of nanobodies in order to leverage their particular abilities in the prevention and treatment of BVDV.

Phage display antibody library technology is an effective means of producing monoclonal antibodies, and it has undergone rapid development in recent years[36]. It can be used to directly clone the genes encoding antibody variable regions in order to facilitate further development of a variety of genetically engineered antibodies[37]. By using this technique, VHH single-domain antibodies produced from phage libraries can be recovered that recognize antigenic epitopes which could not be recognized by other previously tested agents. The construction of the phage antibody library depends on the high variability region of the conventional antibody and VHH recombinant antibody CDRs, which will determine whether the phage antibody will have a great relationship with the antibody library[38]. Conventional IgG antibodies have six antigen complementary binding regions (CDR), while VHH has only three antigen CDRs[39, 40]. The amino acid sequence of VHH featured extended CDR1 and CDR3, which could mitigate deficiencies in antigen binding capacity due to the lack of a light chain[41]. Unlike conventional IgG antibodies, the VHH antigen epitope of CDR3 is formed through convex structure topology. Antigens of this structure combine more readily with the target antigen, such as at the active site of the protease and at the binding sites of the virus and its host cells in order to improve the affinity of the VHH and antigen specificity[42].

## Conclusions

In conclusion, we have shown that nanobody screening through a phage display approach can aid in developing new vaccine technologies and ultimately mitigating BVDV infectivity. Nested PCR was used to amplify the single domain heavy chain antibody variable region sequence from a camel, and a 450 bp sequence was obtained that was consistent with nanobody characteristics. A nanobody library was obtained using helper phage rescue with a capacity of 1.3×10^11^ which indicates that the library capacity was large enough to meet the requirements of phage display technology. After three rounds of affinity screening for the BVDV-E2 protein, five nanobodies that specifically bind the BVDV-E2 protein were obtained and virus replication experiments were carried out using the VHH15 candidate protein which exhibited the best binding characteristics. Results indicated that the nanobody could be used to neutralize BVDV infection of cells, and thus provide a new avenue for targeted drug research and epidemic control of BVDV.

## References

[1] Fu Q, Shi H, Chen C. Roles of bta-miR-29b promoter regions DNA methylation in regulating miR-29b expression and bovine viral diarrhea virus NADL replication in MDBK cells. Arch Virol. 2016. 10.1007/s00705-016-3107-1 27766427

[2] Carta A, Briguglio I, Piras S, Corona P, Ibba R, Laurini E, et al A combined in silico/in?vitro approach unveils common molecular requirements for efficient BVDV RdRp binding of linear aromatic N-polycyclic systems. Eur J Med Chem. 2016;117:321–34. doi: 10.1016/j.ejmech.2016.03.080 27161176 10.1016/j.ejmech.2016.03.080

[3] Alves PA, Figueiredo PO, de Oliveira CH, Barbosa JD, Lima DH, Bomjardim HA, et al Occurrence of Pseudocowpox virus associated to Bovine viral diarrhea virus-1, Brazilian Amazon. Comp Immunol Microbiol Infect Dis. 2016;49:70–5. doi: 10.1016/j.cimid.2016.09.005 27865267 10.1016/j.cimid.2016.09.005

[4] Ridpath JF, Bayles DO, Neill JD, Falkenberg SM, Bauermann FV, Holler L, et al Comparison of the breadth and complexity of bovine viral diarrhea (BVDV) populations circulating in 34 persistently infected cattle generated in one outbreak. Virology. 2015;485(25):297–304. 26319211 10.1016/j.virol.2015.07.022

[5] Downey-Slinker ED, Ridpath JF, Sawyer JE, Skow LC, Herring AD. Antibody titers to vaccination are not predictive of level of protection against a BVDV type 1b challenge in Bos indicus—Bos taurus steers. Vaccine. 2016;34(42):5053–9. doi: 10.1016/j.vaccine.2016.08.087 27601344 10.1016/j.vaccine.2016.08.087

[6] Platt R, Kesl L, Guidarini C, Wang C, Roth JA. Comparison of humoral and T-cell-mediated immune responses to a single dose of Bovela ®; live double deleted BVDV vaccine or to a field BVDV strain. Veterinary Immunology & Immunopathology. 2017. 10.1016/j.vetimm.2017.03.003 28494925

[7] Quinet C, Czaplicki G, Dion E, Dal Pozzo F, Kurz A, Saegerman C. First Results in the Use of Bovine Ear Notch Tag for Bovine Viral Diarrhoea Virus Detection and Genetic Analysis. PLoS One. 2016;11(10):e0164451 doi: 10.1371/journal.pone.0164451 27764130 10.1371/journal.pone.0164451 PMC5072587

[8] Van Engen NK, Platt R, Roth JA, Stock ML, Engelken T, Vann RC, et al Impact of oral meloxicam and long-distance transport on cell-mediated and humoral immune responses in feedlot steers receiving modified live BVDV booster vaccination on arrival. Vet Immunol Immunopathol. 2016;175:42–50. doi: 10.1016/j.vetimm.2016.05.006 27269791 10.1016/j.vetimm.2016.05.006

[9] Workman AM, Heaton MP, Harhay GP, Smith TP, Grotelueschen DM, Sjeklocha D, et al Resolving Bovine viral diarrhea virus subtypes from persistently infected U.S. beef calves with complete genome sequence. J Vet Diagn Invest. 2016;28(5):519–28. doi: 10.1177/1040638716654943 27400958 10.1177/1040638716654943

[10] Zhu L, Lu H, Cao Y, Gai X, Guo C, Liu Y, et al Molecular Characterization of a Novel Bovine Viral Diarrhea Virus Isolate SD-15. PLoS One. 2016;11(10):e0165044 doi: 10.1371/journal.pone.0165044 27764206 10.1371/journal.pone.0165044 PMC5072660

[11] Pecora A, Aguirreburualde MS, Aguirreburualde A, Leunda MR, Odeon A, Chiavenna S, et al Safety and efficacy of an E2 glycoprotein subunit vaccine produced in mammalian cells to prevent experimental infection with bovine viral diarrhoea virus in cattle. Vet Res Commun. 2012;36(3):157–64. doi: 10.1007/s11259-012-9526-x 22639081 10.1007/s11259-012-9526-x

[12] Zhang X, Diraviyam T, Li X, Yao G, Michael A. Preparation of chicken IgY against recombinant E2 protein of bovine viral diarrhea virus (BVDV) and development of ELISA and ICA for BVDV detection. Biosci Biotechnol Biochem. 2016:1–6. 10.1080/09168451.2016.1217144 27484991

[13] Loy JD, Gander J, Mogler M, Vander Veen R, Ridpath J, Harris DH, et al Development and evaluation of a replicon particle vaccine expressing the E2 glycoprotein of bovine viral diarrhea virus (BVDV) in cattle. Virol J. 2013;10(1):35. 23356714 10.1186/1743-422X-10-35 PMC3565941

[14] Cavallaro AS, Mahony D, Commins M, Mahony TJ, Mitter N. Endotoxin-free purification for the isolation of bovine viral diarrhoea virus E2 protein from insoluble inclusion body aggregates. Microb Cell Fact. 2011;10:57 doi: 10.1186/1475-2859-10-57 21787435 10.1186/1475-2859-10-57 PMC3160874

[15] Maya L, Puentes R, Reolón E, Acuña P, Riet F, Rivero R, et al Molecular diversity of bovine viral diarrhea virus in uruguay. Arch Virol. 2016;161(3):529–35. doi: 10.1007/s00705-015-2688-4 26597189 10.1007/s00705-015-2688-4

[16] Desmyter A, Spinelli S, Roussel A, Cambillau C. Camelid nanobodies: killing two birds with one stone. Current Opinion in Structural Biology. 2015;32:1–8. doi: 10.1016/j.sbi.2015.01.001 25614146 10.1016/j.sbi.2015.01.001

[17] Könning D, Zielonka S, Grzeschik J, Empting M, Valldorf B, Krah S, et al Camelid and shark single domain antibodies: structural features and therapeutic potential. Current Opinion in Structural Biology. 2016;45:10–6. doi: 10.1016/j.sbi.2016.10.019 27865111 10.1016/j.sbi.2016.10.019

[18] Roovers RC, Dongen GV. Nanobodies in therapeutic applications. Current Opinion in Molecular Therapeutics. 2007;9(9):327–35. 17694445

[19] Helma J, Cardoso MC, Muyldermans S, Leonhardt H. Nanobodies and recombinant binders in cell biology. Journal of Cell Biology. 2015;209(5):633–44. doi: 10.1083/jcb.201409074 26056137 10.1083/jcb.201409074 PMC4460151

[20] Yan J, Li G, Hu Y, Ou W, Wan Y. Construction of a synthetic phage-displayed Nanobody library with CDR3 regions randomized by trinucleotide cassettes for diagnostic applications. Journal of Translational Medicine. 2014;12(1):343-. 25496223 10.1186/s12967-014-0343-6 PMC4269866

[21] Wesolowski J, Alzogaray V, Reyelt J, Unger M, Juarez K, Urrutia M, et al Single domain antibodies: promising experimental and therapeutic tools in infection and immunity. Medical Microbiology and Immunology. 2009;198(3):157–74. doi: 10.1007/s00430-009-0116-7 19529959 10.1007/s00430-009-0116-7 PMC2714450

[22] Koch K, Wernery U, Khazanehdari K, Danquah W, Koch-Nolte F, Dietrich U. P-D1 Selection and characterization of neutralizing nanobodies from dromedaries immunized with soluble trimeric HIV-1 Env SOSIP proteins. Jaids Journal of Acquired Immune Deficiency Syndromes. 2016;71.

[23] Meyer TD, Muyldermans S, Depicker A. Nanobody-based products as research and diagnostic tools. Trends in Biotechnology. 2014;32(5):263–70. doi: 10.1016/j.tibtech.2014.03.001 24698358 10.1016/j.tibtech.2014.03.001

[24] Mody KT, Mahony D, Cavallaro AS, Zhang J, Zhang B, Mahony TJ, et al Silica Vesicle Nanovaccine Formulations Stimulate Long-Term Immune Responses to the Bovine Viral Diarrhoea Virus E2 Protein. PLoS One. 2015;10(12). 10.1371/journal.pone.0143507 PMC4668082 26630001

[25] Zhang X, Diraviyam T, Li X, Yao G, Michael A. Preparation of chicken IgY against recombinant E2 protein of bovine viral diarrhea virus (BVDV) and development of ELISA and ICA for BVDV detection. Bioscience Biotechnology & Biochemistry. 2016;80(12). 10.1080/09168451.2016.1217144 27484991

[26] Li T, Xu Y, Liu L, Huang M, Wang Z, Tong Z, et al Brucella Melitensis 16M Regulates the Effect of AIR Domain on Inflammatory Factors, Autophagy, and Apoptosis in Mouse Macrophage through the ROS Signaling Pathway. 2016;11(12). doi: 10.1371/journal.pone.0167486 27907115 10.1371/journal.pone.0167486 PMC5132199

[27] Kazemi-Lomedasht F, Behdani M, Bagheri KP, Habibi-Anbouhi M, Abolhassani M, Arezumand R, et al Inhibition of angiogenesis in human endothelial cell using VEGF specific nanobody. Molecular Immunology. 2015;65(1):58–67. doi: 10.1016/j.molimm.2015.01.010 25645505 10.1016/j.molimm.2015.01.010

[28] Gray MA, Tao RN, Deporter SM, Spiegel DA, Mcnaughton B. A Nanobody Activation Immunotherapeutic That Selectively Destroys HER2-Positive Breast Cancer Cells. ChemBioChem. 2016;17(2):155–8. doi: 10.1002/cbic.201500591 26556305 10.1002/cbic.201500591 PMC5199233

[29] Crasson O, Rhazi N, Jacquin O, Freichels A, Ruth N, Galleni M, et al Enzymatic functionalization of a nanobody using protein insertion technology. Protein Engineering, Design and Selection. 2015;28(10). 10.1093/protein/gzv020 25852149

[30] Ju SY, Kyun PS, Jung JY, Na KY, Sung KK, Kyu PO, et al Nanobody-targeted E3-ubiquitin ligase complex degrades nuclear proteins. Scientific Reports. 2015;5. 10.1038/srep14269 PMC4571616 26373678

[31] Bruce VJ, Lopez-Islas M, Mcnaughton BR. Resurfaced cell-penetrating nanobodies: A potentially general scaffold for intracellularly targeted protein discovery. Protein Science. 2016;25(6):1129–37. doi: 10.1002/pro.2926 26991318 10.1002/pro.2926 PMC4941773

[32] Sayers RG, Sayers GP, Graham DA, Arkins S. Impact of three inactivated bovine viral diarrhoea virus vaccines on bulk milk p80 (NS3) ELISA test results in dairy herds. Veterinary Journal. 2015;205(1):56. 10.1016/j.tvjl.2015.03.025 25986132

[33] Braun MB, Traenkle B, Koch PA, Emele F, Weiss F, Poetz O, et al Peptides in headlock–a novel high-affinity and versatile peptide-binding nanobody for proteomics and microscopy. Scientific Reports. 2016;6:19211 doi: 10.1038/srep19211 26791954 10.1038/srep19211 PMC4726124

[34] El-Attar LMR, Thomas C, Luke J, Williams JA, Brownlie J. Enhanced neutralising antibody response to bovine viral diarrhoea virus (BVDV) induced by DNA vaccination in calves. Vaccine. 2015;33(32):4004 doi: 10.1016/j.vaccine.2015.06.017 26079613 10.1016/j.vaccine.2015.06.017

[35] Ciulli S, Galletti E, De GF, Battilani M, Prosperi S. The use of SSCP analysis [corrected] to evidence genetic variability in the gene coding for immunodominant protein e2 of the BVDV. Veterinary Research Communications. 2008;32(1):183–5. 10.1007/s11259-008-9164-5 18683063

[36] Zhao A, Tohidkia MR, Siegel DL, Coukos G, Omidi Y. Phage antibody display libraries: a powerful antibody discovery platform for immunotherapy. Critical Reviews in Biotechnology. 2016;36(2):1–14. 25394539 10.3109/07388551.2014.958978

[37] Valadan R, Rafiei A, Hashemitabr G, Bassami MR. Protein a-specific enrichment of insert-containing phage in antibody phage display library. Journal of Mazandaran University of Medical Sciences. 2015;24(120):43–53.

[38] Negi P, Lövgren J, Malmi P, Sirkka N, Metso J, Huovinen T, et al Identification and analysis of anti-HDL scFv-antibodies obtained from phage display based synthetic antibody library. Clinical Biochemistry. 2015;53(4). 10.1016/j.clinbiochem.2015.11.020 26656638

[39] Kingston HWF, Blacksell SD, Tanganuchitcharnchai A, Laongnualpanich A, Basnyat B, Day NPJ, et al Comparative accuracy of the InBios Scrub Typhus Detect™ IgM Rapid Test for the detection of IgM antibodies using conventional serology. Clinical & Vaccine Immunology Cvi. 2015;99(10):1130–2. 10.1128/CVI.00390-15 PMC4580738 26291089

[40] Akazawa-Ogawa Y, Uegaki K, Hagihara Y. The role of intra-domain disulfide bonds in heat-induced irreversible denaturation of camelid single domain VHH antibodies. The Journal of Biochemistry. 2016;159(1). 10.1093/jb/mvv082 PMC4882646 26289739

[41] Shkoporov AN, Khokhlova EV, Savochkin KA, Kafarskaia LI, Efimov BA. Production of biologically active scFv and VHH antibody fragments in Bifidobacterium longum. FEMS Microbiology Letters. 2015;362(12). 10.1093/femsle/fnv083 25994292

[42] Nakayama H, Murakami A, Yoshida M, Muraoka J, Wakai J, Kenjyou N, et al Characterization and Selection of 3-(1-Naphthoyl)-Indole Derivative-Specific Alpaca VHH Antibodies Using a Phage Display Library. Monoclonal Antibodies in Immunodiagnosis & Immunotherapy. 2016;35(4):231–4. 27556911 10.1089/mab.2016.0003

